# Exploring the Role of Rhizobacteria in *Sorghum bicolor* Adaptation to Combined Drought and Heat Stress

**DOI:** 10.3390/microorganisms13112454

**Published:** 2025-10-26

**Authors:** Alec Magaisa, Elizabeth Ngadze, Tshifhiwa Paris Mamphogoro, Martin Philani Moyo, Casper Nyaradzai Kamutando

**Affiliations:** 1 Department of Plant Production Sciences & Technologies, University of Zimbabwe, Mt Pleasant, Harare P.O. Box MP 167, Zimbabwe; alecmagaisa@gmail.com (A.M.); ngadzee@gmail.com (E.N.); 2 International Crops Research Institute for the Semi-Arid Tropics, Matopos Research Station, Bulawayo P.O. Box 776, Zimbabwe; martin.moyo@icrisat.org; 3 Gastro-Intestinal Microbiology and Biotechnology Unit, Agricultural Research Council-Animal Production, Private Bag X02, Irene, Pretoria 0062, South Africa; mamphogorot@arc.agric.za

**Keywords:** rhizomicrobiota, novel breeding technique, abiotic stress tolerance

## Abstract

Although rhizobacteria are known to improve plant adaptation to abiotic stressors, their possible contribution to the inherent resilience exhibited by crops such as *Sorghum bicolor* is still poorly quantified. Here, three sorghum pre-release lines and three check varieties were established and evaluated at two low-altitude sites of less than 600 masl. Treatments were laid out in a randomized complete block design, replicated two times. Twenty-four rhizospheric soil samples comprising six sorghum genotypes with two replications across two sites were collected, processed using Zymo Research DNA extraction protocols, and the 16S rRNA amplicon sequences were generated for bacterial diversity quantifications following the Divisive Amplicon Denoising Algorithm 2 (DADA2) workflow. Grain yield data were also recorded and expressed in tonnes per hectare. Rhizobacteria recruitment and GY performance significantly (*p* < 0.05) varied with sorghum genotypes. Bacterial abundance significantly (*p* < 0.05) associated with sorghum grain yield performance with *Actinobacteriota* and *Firmicutes* being identified to be of economic importance, explaining between 52.23 and 85.64% of the variation in grain yield performance. The modelled relationships between rhizobacteria and grain yield performance revealed R^2^ predicted values of up to 75.25% and a 10-fold R^2^ of 75.54%, implying no model overfitting. Sorghum genotypes did not consistently exhibit direct variation between genetic worth values and grain yield performance. Superior grain yield performers, namely ICSV111IN, CHITICHI, and SV4, consistently associated with high incidences of occurrence of the bacteria phyla *Chloroflexi* (class = *Chloroflexia*) and Firmicutes (class = *Bacilli*), whilst integrating the conventional selection method with microbial diversity data, changed the genotype performance ranking, in which all the three pre-release lines, namely, IESV91070DL, ASARECA12-3-1, and ICSV111IN, exhibited superiority over the check varieties. The results demonstrated that the inherent stress resilience exhibited by some sorghum genotypes under climate change-induced stresses such as CDHS may be influenced by specific bacterial taxa recruited in the rhizosphere environment of the plants. Hence, more effort should be made to further exploit these beneficial plant–microbe interactions for enhanced sorghum productivity under abiotic stress conditions.

## 1. Introduction

Abiotic stress, specifically combined drought and heat stress (CDHS), continues to negatively impact agriculture [1], causing an estimated 33% loss in total production for cereal crops globally [2]. This presents a serious challenge given the projections that by the year 2050, food production should have increased from 60 to 100% to match the increases in population globally [3]. Whilst conventional breeding methods have been used to improve crop varieties [4,5], not all abiotic factors can be addressed through these methods [6]. Drought and heat are natural plant challenges [6], whilst plants and their associated microorganisms are considered a single evolutionary unit [7]. As such, utilizing rhizomicrobiota in crop improvement could be an effective nature-based breeding strategy against these abiotic stressors [8].

Exploring the use of rhizomicrobiota in abiotic stress management could be a possibility given that plants are known to recruit beneficial rhizosphere microorganisms for improved abiotic stress tolerance [9,10,11]. This microbial recruitment follows a “cry for help” mechanism triggered by plants when subjected to abiotic stress, forming symbiotic partnerships with plants for survival under harsh environments [12,13]. The plant and microbiome interactions occur in the rhizosphere. The rhizosphere is defined as the soil zone that surrounds and is influenced by plant roots, an active zone of interaction between plants and soil microbiome [14,15]. It is a vital microbial hotspot or an ecological interface between plants and soil microorganisms, a self-adjusting system kind of “gut–brain axis” in plants [14].

These eco-evolutionary interactions between plant hosts and their associated microbiomes are projected to be of paramount importance in addressing climate change problems, particularly combined drought and heat stress [15]. This is based on the premise that plants and their microbiome coexisted and coevolved for many years [16], and they must have devised mechanisms to fight for each other under danger [16,17,18]. As a result, there has been a growing interest in how rhizomicrobiota can be utilized in managing abiotic stresses [16]. Breeding programmes can aim to identify the beneficial soil microorganisms for use as plant inoculants against CDHS [12,19,20]. However, before such actions, there is a need for further research to enhance understanding of how plant–microbiome interactions occur in the rhizosphere to cause impact crop productivity.

Whilst sorghum is a resilient crop, it is not completely immune to abiotic stressors [20]. For instance, significant variation has been reported in sorghum’s response to severe drought and heat stresses, especially when the stresses occur during the pre- and post-flowering stages [4]. Therefore, it could be a scientific misconception to entirely attribute sorghum abiotic stress tolerance to its genetic potential, responsible for the sustenance of all the biochemical and physiological processes that drive plant growth and development. Here, we hypothesize that sorghum genotypes have variable levels of stress resilience, aided by the kind of beneficial microorganisms they recruit into the rhizosphere, where the level of abiotic stress tolerance is manifested through grain yield performance. Therefore, this study aims to depict the possible contributions of rhizobacteria in conferring sorghum adaptation to combined drought and heat stress conditions. The study specifically sought to achieve the following: (i) determining if rhizobacteria recruitment differs with *Sorghum bicolor* genotypes; (ii) quantifying the impact of rhizobacteria on GY performance of *Sorghum bicolor* genotypes under CDHS conditions; and (iii) assessing the applicability of utilizing rhizobacteria for their traits plant selection to develop climate-resilient crop varieties. This particular study was anchored on the “cry for help” mechanism triggered by plants when subjected to stress conditions to then expect that certain *Sorghum bicolor* genotypes recruit specific beneficial rhizobacteria for tolerance against CDHS where plant response is manifested in the form of GY performance.

## 2. Materials and Methods

### 2.1. Planting Materials, Experimental Design, Study Sites, and Crop Management

**Figure 1:**
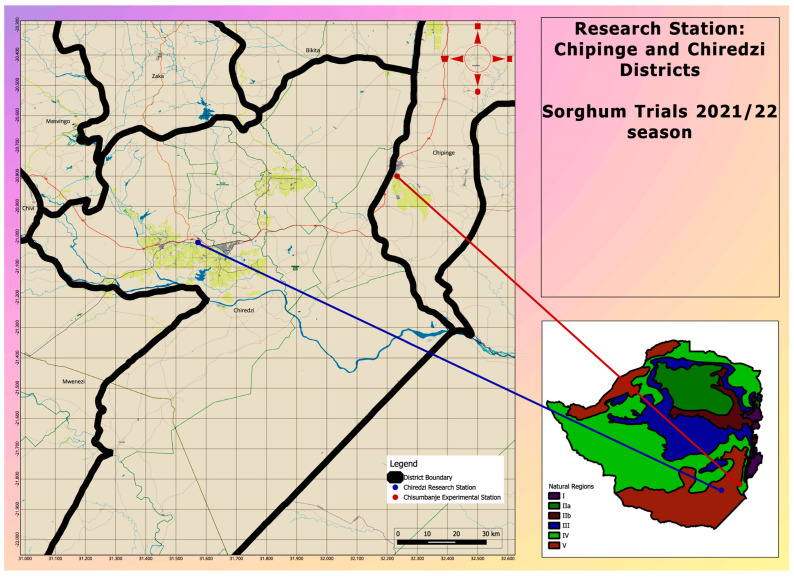
A map indicating the location of study sites where *Sorghum bicolor* genotypes were evaluated during the 2021–22 season under CDHS conditions.

Three advanced pre-release *Sorghum bicolor* lines were established together with three check varieties (Table 1), at two sites, both located at low-altitude (i.e., <600 masl) zones, in the south-eastern lowveld of Zimbabwe (Figure 1). The two sites were selected because of their long-term history of very high temperatures at the beginning of the summer season [21]. At each site, the six treatments were laid out under open-field conditions, using a randomized complete block design (RCBD), with two replications.

Planting was performed by dribbling seeds in furrows of 5 m, four-row plots, with 0.75 m inter-row spacing. After three weeks, post-emergence thinning was performed to achieve 0.2 m in-row spacing, meaning 100 plants per plot. Grain yield (GY) was measured from the grain collected from the heads of plants from the middle two rows of each plot; a 0.5 m border on each end of the rows was discarded to eliminate border effects. This resulted in a net plot size of 9 m^2^ (i.e., 3 rows × 0.75 m × 4 m row length), which was equivalent to a population of 40 plants per plot. At the early vegetative stage, genotypes were subjected to CDHS conditions created by withholding supplementary irrigation for two weeks during the hottest periods in September and October, when average daily temperatures exceed 30 degrees Celsius [21]. The CDHS field conditions were characterized by average high temperatures of 38 degrees Celsius and average low temperatures of 20 degrees Celsius during a dry season that could only sustain crop production under irrigation.

### 2.2. Data Collection

#### 2.2.1. Rhizospheric Soil Sampling for 16S rRNA Amplicon Sequencing

A total of 24 rhizospheric soil samples (i.e., 6 sorghum genotypes × 2 replications × 2 sites) were collected. At each plot, the rhizospheric soil sampling process involved the manual extraction of three whole plants with their root systems, followed by the removal of the core soil, performed by hitting and shaking plant roots and then thoroughly mixing to prepare a composite soil sample. From each composite sample, 200 g was sub-sampled for 16S rRNA amplicon sequencing at Inqaba Biotechnical Industries in Pretoria, South Africa (www.inqababiotec.co.za, accessed on 27 November 2021).

Briefly, genomic DNA was extracted using Quick DNA Fecal/Soil Microbe MiniPrep™ Kit (Zymo Research, Irvine, CA, USA) following the manufacturer’s protocol: https://zymoresearch.eu/products/quick-dna-fecal-soil-microbe-dna-miniprep-kit, accessed on 27 November 2021. Polymerase Chain Reaction (PCR) was used to amplify the genomic DNA samples using a universal primer pair, 515F and 806R, targeting the V4 region of the bacterial 16S rRNA gene [22]. The resultant amplicons were purified and end-repaired; they were then ligated using Illumina-specific adapter sequences (NEBNext Ultra II DNA library prep kit, New England Biolabs, Ipswich, MA, USA). Following quantification, the samples were individually indexed (NEBNext Multiplex Oligos for Illumina Dual Index Primers Set 1, New England Biolabs, Ipswich, MA, USA), and another AMPure XP bead-based purification step was performed. Amplicons were then sequenced on the Illumina’s NextSeq500 platform, using a NextSeq (300-cycle) kit (Illumina, San Diego, CA, USA). Approximately, 20 Mb of data (2 × 150 bp long paired-end reads) were produced for each sample. A platform setting was used to remove adapters post-sequencing.

#### 2.2.2. Grain Yield Performance

After the threshing of sorghum heads per net plot area, GY performance data were collected and expressed in tonnes per hectare using the following formula:

$$Grain\;yield\;(t/{ha}^{-1})=1000\times \frac{Net\;plot\;grain\;weight\;(kg)}{Net\;plot\;size\;(m^{2})/10,000}$$

### 2.3. Data Analysis

#### 2.3.1. 16S rRNA Sequence Analysis

**Figure 2:**
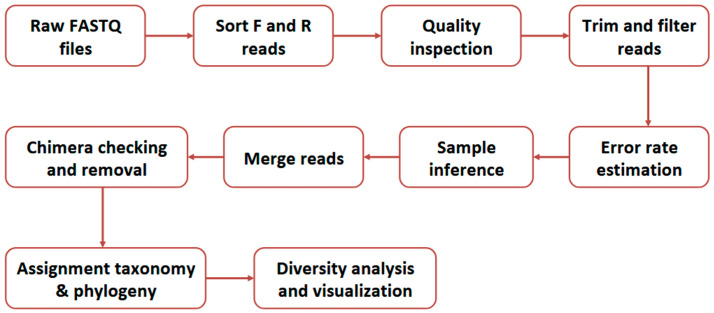
The summary outline of the Divisive Amplicon Denoising Algorithm 2 (DADA2) workflow [23].

The 16S rRNA sequencing data were analyzed following the Divisive Amplicon Denoising Algorithm 2 (DADA2) workflow (see Figure 2) using the phyloseq v4.2.3 R package [23].

#### 2.3.2. Bacteria Diversity Analysis

The Kruskal–Wallis Test was deployed to test for statistical differences in Alpha diversity or the within-sample diversity based on the Chao1 Index. Permutational multivariate analysis of variance (PERMANOVA) was used to assess Beta diversity, thus tracking differences in microbial community composition between the two study sites based on Bray–Curtis dissimilarity [24]. Both the PERMANOVA analysis and the Chao1 Alpha diversity index estimation were performed using the R v3.4.3 “vegan” package [23].

#### 2.3.3. Rhizobacteria Incidence of Occurrence and Their Impact on *Sorghum bicolor* GY Performance

The Poisson regression was deployed to quantify the likelihood (probabilities) of the occurrence of specific bacterial taxa in association with certain *Sorghum bicolor* genotypes. The Poisson probability analysis was selected for application because of its suitability in modelling count or rare events data [25]. The estimation of those rate ratios enabled researchers to track the linkages between microbial counts or occurrence and plant host GY performance.

Multiple linear regression (MLR) modelling was performed to establish and quantify the relationships between rhizobacteria abundance and *Sorghum bicolor* GY performance, also identifying the rhizobacteria of importance in sorghum bicolor tolerance to CDHS. Both the Poisson and multiple linear regression (MLR) analyses used the 988-rhizoplane operational taxonomic units (OTUs) with a 97% occurrence in all samples (common OTUs) as input data whilst the Z-score Standardization method was used for data normalization. Both techniques were applied using Minitab Statistical Software Version 21.4. [26,27], in which the coefficient of determination (R^2^) was used as an effect size measure in regression modelling [28]. Furthermore, the K-fold validation, R^2^ predicted, and BIC were utilized as model validation metrics. In this study, it was important and applicable to predict or model the spatiotemporal dynamics of rhizobacteria communities and to quantify their impact on sorghum bicolor GY performance [16]. The 24 rhizospheric soil samples for DNA sequencing were collected at both pre-flowering (vegetative) and post-flowering (reproductive) growth stages. However, statistical analysis tracking the impact of rhizobacteria on sorghum GY performance focused on data collected at the post-flowering growth stage, at which the crop is reportedly more susceptible to abiotic stress.

#### 2.3.4. Integrating Microbial Diversity Indices to Select Superior *Sorghum bicolor* Genotypes for Production Under CDHS Conditions

Using the observed rhizobacteria as targeted traits of importance in plant selection, the Smith–Hazel Multi-Trait Stability Index (MTSI) analysis was used to select superior *Sorghum bicolor* genotypes based on their genetic worths [29].

The genetic worth *I* of an individual *Sorghum bicolor* genotype based on traits *x*, *y*, …, *n*, is calculated as follows:

$$I=\;b_{x}G_{x}+\;b_{y}\;G_{y}+\ldots +\;b_{n}G_{n}$$

where b is the index coefficient for the traits x, y, …, n, respectively, and G is the individual genotype BLUPs for the traits x, y, …, n, respectively.

It was anticipated that rhizobacteria rarely operate in isolation but collectively influence host plant adaptation to CDHS conditions, hence the application of the Smith–Hazel Multi-Trait Stability Index (MTSI) analysis.

## 3. Results

### 3.1. Bacterial Diversity and Community Composition

**Figure 3:**
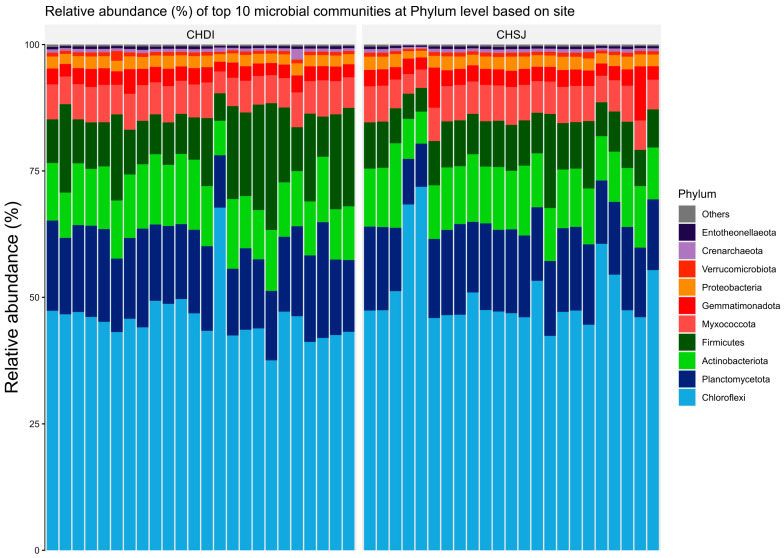
Relative abundance (%) of the top 10 bacterial phyla, associated with the rhizosphere of sorghum at two low-altitude sites, namely Chiredzi Research Station (CHDI) and Chisumbanje Research Station (CHSJ), in the south-eastern lowveld of Zimbabwe.

**Figure 4:**
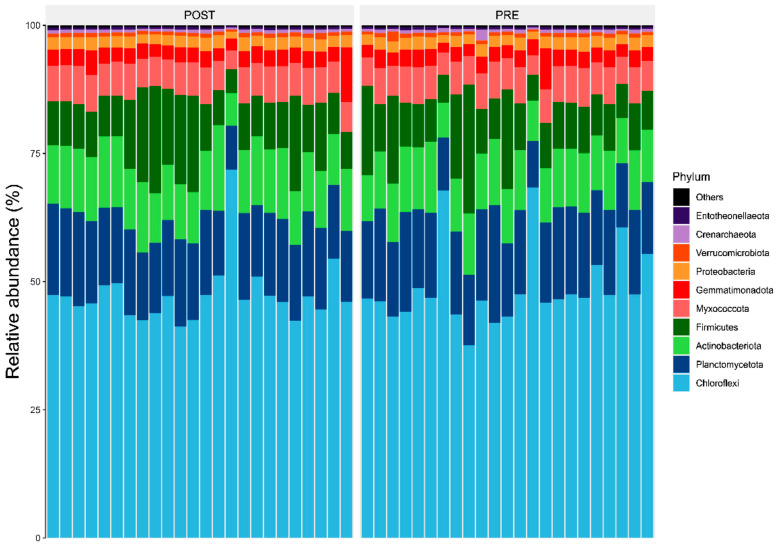
Relative abundance (%) of the top 10 bacterial phyla, associated with the rhizosphere of sorghum during its pre- and post-flowering growth stages, under combined drought and heat stress (CDHS) conditions. PRE refers to pre-flowering or vegetative crop growth stage whilst POST is post-flowering or crop reproductive growth stage at which rhizospheric soil samples were collected for DNA sequencing to conduct microbial community composition analysis.

Results showed members of the *Chloroflexi*, *Firmicutes*, and *Actinobacteriota* were the most abundant bacterial phyla associated with sorghum roots across the two sites (i.e., Chisumbanje and Chiredzi) (Figure 3). In addition, the same bacterial phyla (i.e., *Chloroflexi*, *Firmicutes* and *Actinobacteriota*) were also identified as the most abundant bacteria associated with the rhizosphere of sorghum during the pre- and post-flowering growth stages (Figure 4).

**Figure 5:**
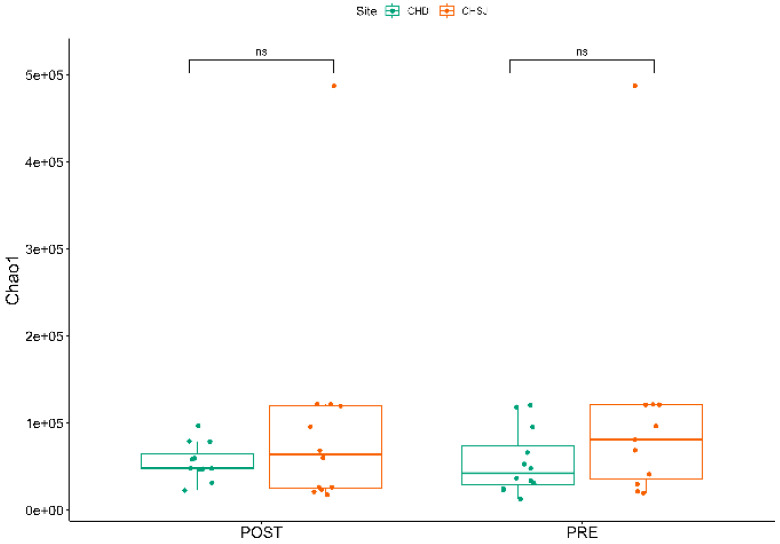
α diversity indicating the richness of bacterial species, associated with the rhizosphere of sorghum genotypes established at two low-altitude sites, i.e., Chiredzi Research Station (CHD) and Chisumbanje Research Station (C~S), at pre- and post-flowering stages of sorghum growth. PRE refers to pre-flowering or vegetative crop growth stage, whilst POST is post-flowering or crop reproductive growth stage at which rhizospheric soil samples were collected for DNA sequencing to conduct microbial community composition analysis. The notation “ns” represents no significant difference at 5% probability level.

There was no significant Alpha diversity across the two study sites and crop growth stages (Figure 5).

**Figure 6:**
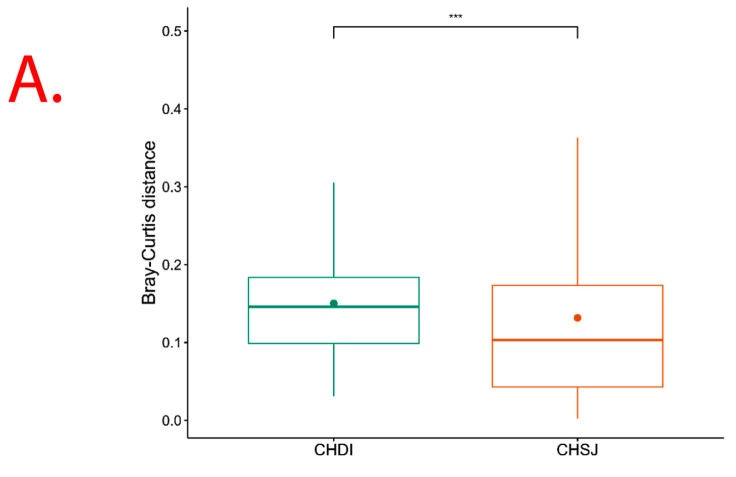

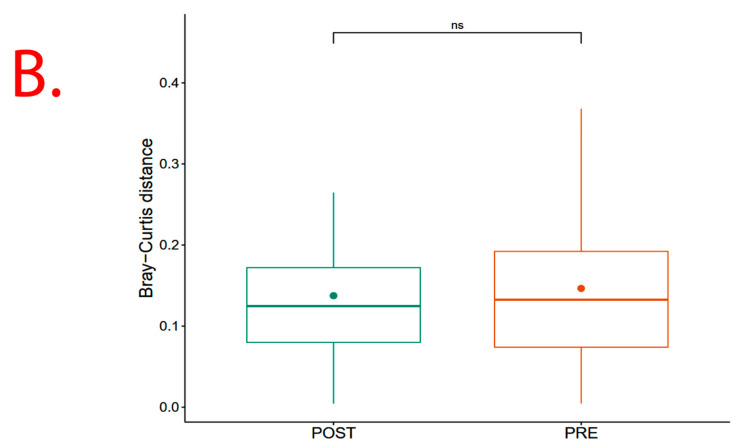
β diversity indicating bacterial species richness associated with the rhizosphere of sorghum: (**A**) across the two low-altitude sites; and (**B**) at pre- and post-flowering crop growth stages. PRE refers to pre-flowering or vegetative crop growth stage, whilst POST is post-flowering or crop reproductive growth stage at which rhizospheric soil samples were collected for DNA sequencing to conduct microbial community composition analysis. CHDI represents Chiredzi Research Station, whereas CHSJ represents Chisumbanje Research Station. This was determined through the permutational multivariate analysis of variance (PERMANOVA). The notations “***’’ and “ns” represent significant and no significant difference at 5% probability level respectively.

However, results show significant (*p* = 0.013) Beta diversity across study sites (i.e., Chisumbanje and Chiredzi), with sites explaining 2.69% of the variation in Bray–Curtis distances (Figure 6). There was no significant (*p* = 0.526) Beta diversity between pre- and post-flowering crop growth stages, with crop growth stages explaining 2.24% of the variation in Bray–Curtis distances (Figure 6).

### 3.2. Rhizobacteria Incidence of Occurrence and Their Impact on Sorghum bicolor GY Performance Under CDHS Conditions

**Figure 7:**
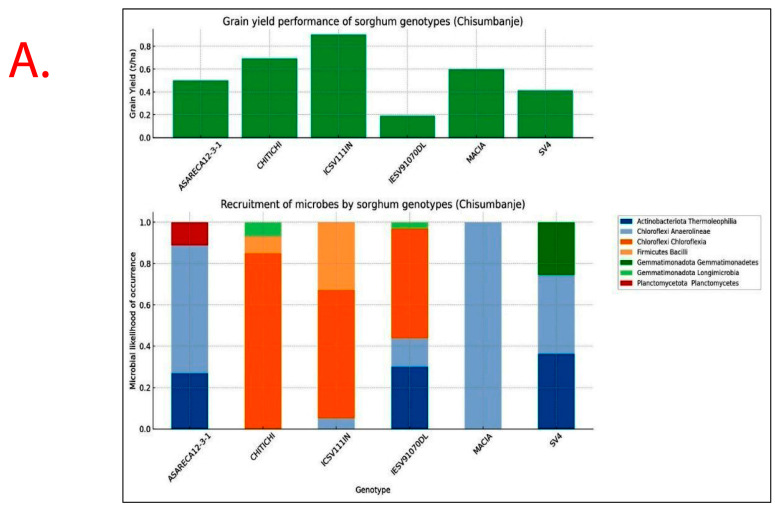

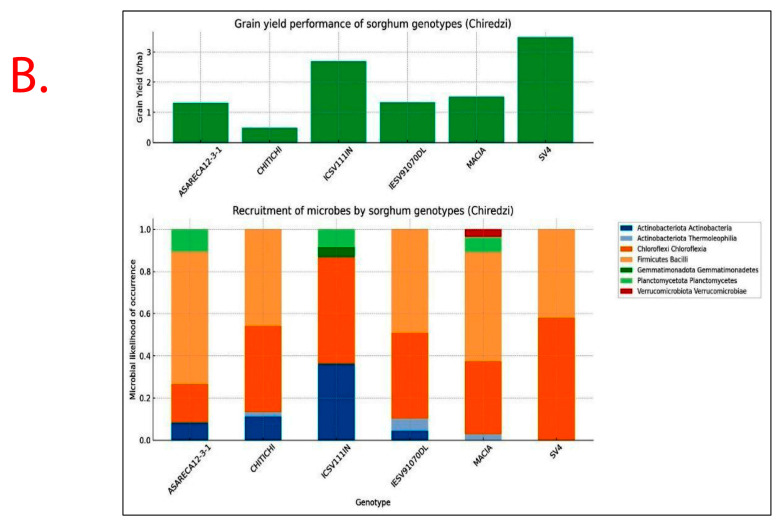
Rhizobacteria likelihood of occurrence with *Sorghum bicolor,* indicating differential levels of microbial recruitment, and the GY performance of sorghum genotypes, established and evaluated at the (**A**) Chisumbanje Research Station and (**B**) Chiredzi Research station during the 2021–22 season under CDHS conditions. The likelihood of occurrence is a Poisson probability computed based on microbial relative abundance data. The taxonomy information is at provides at phylum and class levels.

Recruitment of bacterial taxa and GY performance significantly varied (*p* < 0.05) with *Sorghum bicolor* genotypes exhibiting specificity and variations across study sites (Figure 7). Sorghum genotypes exhibiting superior GY performance, i.e., ICSV111IN and CHITICHI (at Chisumbanje Research Station), and SV4 and ICSV111IN (at Chiredzi Research Station), were consistently associated with the high occurrence of the members of the bacterial class *Chloroflexia* (phylum = *Chloroflexi*) and to some extent *Bacilli* (phylum = *Firmicutes*) (Figure 7).

Rhizobacteria relative abundances were significantly (*p* = 0.000) associated with *Sorghum bicolor* GY performance at Chisumbanje Research Station, with bacterial phylum *Actinobacteriota* (class = *Thermoleophilia*) and *Firmicutes* (class = *Bacilli*) explaining 85.64% of the variation in GY performance (Table 2). The modelled relationship revealed 75.25% predictive power and a 75.54% 10-fold R^2^.

At Chiredzi Research Station, rhizobacteria were also significantly (*p* = 0.036) associated with sorghum genotypes’ GY performance, attaining a 52.23% collective explanatory power, contributed differentially by members of the bacterial phyla *Firmicutes* and *Actinobacteriota* (Table 3).

### 3.3. Integrating Microbial Diversity Indices to Select Superior Sorghum bicolor Genotypes for Production Under CDHS Conditions

Sorghum genotypes revealed significant (*p* < 0.05) GY performance, also showing differential breeding values based on the estimated Smith–Hazel genetic worth indices (Table 4).

The ranking of *Sorghum bicolor* genotypes changed between the selection criteria, viz, under conventional selection based on GY performance and under the conventional method integrated with microbial diversity data (i.e., GY performance and genetic worth) (Table 5).

## 4. Discussion

Subjected to danger, every organism on earth tends to rely on its neighbours for survival [30]; this applies to plants with rhizobacteria under abiotic stressors. From an ecological, evolutionary, and plant breeding perspective, it could be catastrophic to assume these plant–microbiome partnerships occur at random. As for sorghum and other plants, there is still a lack of knowledge on how rhizobacteria can be utilized in breeding as a strategy to develop climate-resilient crop varieties [4]. This study, therefore, aims to showcase the possible contributions or roles rhizobacteria play in promoting *Sorghum bicolor* adaptation to CDHS. The study was anchored on the hypothesis that certain *Sorghum bicolor* genotypes selectively recruit beneficial bacteria into their rhizosphere for enhanced adaptation to CDHS, where the level of stress tolerance is manifested in the form of GY performance.

The variation in rhizobacteria recruitment with *Sorghum bicolor* genotypes, resulting in differential GY performance (Figure 7), indicates the presence of genotypic variation, which can be exploited in crop improvement programmes to develop climate-resilient varieties. This finding suggests that the observed coexistence of *Sorghum bicolor* genotypes with rhizobacteria under CDHS conditions cannot be attributed to chance [30]. Instead, evidence suggest plant and rhizobacteria association being a systematically modulated process. In support of this finding, previous studies reported that, when plants are subjected to abiotic stressors, they have the ability to produce exudates in their roots, which act as either repellents or attractants of specific plant growth-promoting microbes [31,32]. It is further reported that rhizosphere microbes are not mere spectators in the recruitment process as they can deploy strategies to also select microbial species to partner with in conferring plant adaptation to abiotic stresses [33,34].

Tracking the impact of rhizobacteria on crop performance under CDHS conditions, we explored the linkages between the microbial incidence of occurrence and *Sorghum bicolor* GY performance (Figure 7). Through regression analysis, we established and tested the association between rhizobacteria abundance and *Sorghum bicolor* GY performance (Table 2 and Table 3).

Results show that high GY performers, i.e., ICSV111IN (pre-release line) and CHITICHI) at the Chisumbanje Research Station (Figure 7) and SV4 and ICSV111IN at the Chiredzi Research Station (Figure 7), were consistently associated with a high incidence of occurrences for members of the bacterial phylum *Chloroflexi* (class = *Chloroflexia*) and *Firmicutes* (class = *Bacilli*). The big question is whether this association was by chance or not. Previous studies report that the association and impact of rhizosphere microbiomes on crop performance under abiotic stress is a result of a specifically controlled symbiotic relationships [32]. Even though members of the bacterial phylum *Chloroflexi* (class = *Chloroflexia*) and *Firmicutes* (class = *Bacilli*) revealed high abundance (Figure 3 and Figure 4), and despite the known fact that “everything is everywhere but the environment selects” [35], results from this study suggest a different story. Rhizobacteria recruitment significantly varied with sorghum genotypes at Chisumbanje Research Station (*p* = 0.0334) and also at Chiredzi Research Station (*p* < 0.0001), and this genotypic variation can be exploited in plant breeding. This finding is in agreement with previous studies, where it was found that various genotypes of the same plant species had distinct microbiome compositions indicating that the microbiome is shaped by host genetics [36].

To further support this, microbial abundance significantly explained variation in sorghum genotypes’ GY performance, exhibiting high explanatory and predictive powers, i.e., R^2^ = 85.64%, R^2^ predicted = 75.25% and a 75.54% 10-fold R^2^ at Chisumbanje (Table 2) and R^2^ = 52.23% at Chiredzi (Table 3). However, study findings suggest that rhizobacteria do not impact crop performance in the same way under CDHS conditions. The modelled relationships (Table 2 and Table 3) identified members of the bacterial phylum *Actinobacteriota* (class = *Thermoleophilia*), *Firmicutes* (class = *Bacilli*) and *Actinobacteriota* (class = *Actinobacteria*) as of economic importance in accounting for the variation in *Sorghum bicolor* GY performance.

Whilst it can be argued that the observed trends cannot conclusively suggest the presence of a cause-and-effect relationship, they could still offer practical plant breeding insights. A 10-fold R^2^ of 75.54% implies no overfitting, indicating that the model generalizes well to unseen data. It suggests that the modelled relationship is valid and could be depended upon in informing plant selection decisions. Members of the *Actinobacteriota Thermoleophilia*, *Firmicutes Bacilli* and *Actinobacteriota Actinobacteria* phyla have been identified to be of paramount importance in enhancing *Sorghum bicolor* adaptation to CDHS. This is in agreement with previous studies, which identified members of the phylum *Firmicutes* and *Actinobacteria* as renowned nitrogen fixers and phosphate-mobilizing microbes [37,38]. Therefore, they help the host plant survive CDHS conditions by changing the atmospheric form of nitrogen into nitrates and insoluble phosphorus into a soluble form for enhanced plant access.

In addition, members of the phylum *Firmicutes* and *Actinobacteriota* are known to secrete the enzyme 1-aminocyclopropane-1-carboxylate deaminase, which converts ACC into ammonia and α-ketobutyrate, thus lowering ethylene levels ultimately promoting growth under abiotic stress conditions [39]. A related study also found members of the *Actinobacteria* being dominant plant root residents in *Sorghum bicolor* [3], leading to increases in the production of hormones specifically GA, IAA, SA, JA, CKs, and BR, which conferred abiotic stress tolerance [20]. Overall, previous studies are in support of the evidence generated from this study, showing that rhizobacteria enhanced *Sorghum bicolor* survival and productivity under CDHS.

Rhizosphere microbes differentially accounting for the variation in *Sorghum bicolor* GY performance across sites could be expected (Table 2 and Table 3). The PERMANOVA analysis showed significant (*p* = 0.013) Beta diversity (Figure 6), indicating differences in bacterial community composition across sites. With differences in bacterial communities available for recruitment across sites, the trends observed in which rhizobacteria differentially impact *Sorghum bicolor* GY performance across study sites make practical and scientific sense.

Although microbial community composition significantly differed across study sites as per PERMANOVA analysis, sorghum genotypes exhibiting superior GY performance, i.e., ICSV111IN and CHITICHI at the Chisumbanje Research Station, and, SV4 and ICSV111IN at the Chiredzi Research Station, were consistently associated with high incidences of occurrence of the members of the bacterial phylum Chloroflexi (class = *Chloroflexia*) and Firmicutes (class = *Bacilli*) (Figure 7). This buttresses the point that plant–microbiome interactions are specifically and systematically controlled processes. However, the insignificant difference in Beta diversity between the pre- and post-flowering growth stages (Figure 6) could suggest that the time of sampling is not of critical importance. Breeders may collect microbial information at either pre- or post-flowering crop growth stages without compromising plant selection decisions.

Evidence from this study also suggests that high microbial abundance does not necessarily imply improved crop performance under CDHS conditions. Although members of the bacterial phylum *Chloroflexi* (class = *Chloroflexia*) were the most abundant (Figure 3 and Figure 4) and showed high likelihood of occurrence with sorghum genotypes of high GY performance (Figure 7), they were not identified as being of economic importance in the modelled relationships between rhizobacteria and GY performance (Table 2 and Table 3). This finding suggests that everything about plant adaptation to CDHS depends upon host plant genetics and microbial factors.

Based on the results from the Smith–Hazel analysis, it is intriguing that not all sorghum genotypes that exhibited high genetic worth revealed GY superiority (Table 5). For instance, at Chisumbanje Research Station, the pre-release genotype IESV91070DL attained the least GY (0.191t/ha) despite it recording the highest genetic worth value (816.660), and similar trends were also observed at the Chiredzi Research Station (Table 5). With the current conventional breeding methods where plant selection is mainly based on aboveground traits like grain yield [5], this pre-release line IESV91070DL would likely be downgraded. Despite their low GY performance, breeding materials exhibiting superior genetic worth could still offer opportunities for further exploitation. Integrating conventional selection method with microbial diversity data introduced an interesting plant selection dimension. The same pre-release line IESV91070DL, potentially of low ranking under conventional selection method based on GY performance, became the winning candidate. Blindly recommending the production or release of crop varieties solely on high GY performance, therefore, risks losing crucial breeding information and could present problems of genetic loss and reduced yields in the future [40].

The low adoption of improved sorghum varieties remains a challenge as farmers continue to rely on their traditional varieties [41]. This could suggest the presence of a plant selection problem calling for remedial action. In a case where sorghum varieties are released solely on high GY performance ignoring their low genetic worth status, it may result in failure to sustain that superiority over time, leading to low adoption. In this study, the traditional variety, CHITICHI, exhibited substantial genetic worth (Table 5), which could translate to stable GY performance over time. This then probably explains why in some instances, farmers would favour landraces over improved sorghum varieties. To address these challenges, we believe there is a need for a paradigm shift in plant breeding thinking and approaches, and to explore the use of rhizobacteria in breeding. The integration of rhizobacteria in current breeding approaches could improve breeding efficiency which also enhancing accelerated crop improvement.

To fully exploit the genetic potential and enhance sustainable adoption of *Sorghum bicolor* varieties, we therefore propose a plant selection strategy that optimizes both GY and genetic worth (Table 5). In this approach, superior genotypes are those that exhibit high average HI and GY values, viz. high average sum of genetic worth and grain yield performance, respectively. The proposed plant selection strategy is not a completely new innovation but an integration of rhizobacteria into the current plant selection methods. Here, rhizobacteria act as the targeted selection traits and main actors in plant adaptation to abiotic stressors and we utilize the Smith–Hazel analysis to estimate their individual genetic merits and the ultimate genetic worth of sorghum genotype.

Members of the *Chloroflexi*, *Actinobacteria* and *Firmicutes* being shown to be the most abundant is consistent with previous studies [37]. It is reported that members of the Firmicutes and Actinobacteria are Gram-positive [37,42], which possess thick cell walls and therefore have the ability to form spores which enable them to withstand the CDHS conditions [43]. It is further reported that the diversity of rhizobacteria decreases during drought treatment [42], whilst the spore-forming ability of *Firmicutes* and *Actinobacteria* ensures that their adaptability to harsh conditions is in place [42,44].

Overall, the evidence generated is compellingly valid considering consistency in the observed trends, agreement with previous studies, and practical significance. However, with improved funding, this same experiment could still be conducted over seasons as well as under controlled environments (i.e., greenhouses and laboratory) in order to further validate the findings.

## 5. Conclusions

Whilst *Sorghum bicolor* has been known to be a resilient crop, its adaptation has generally been attributed to other factors not rhizobacteria. However, this study effectively demonstrated and quantified the role rhizobacteria potentially play in sorghum adaptation to CDHS. Results from this study suggest that the inherent stress resilience exhibited by some sorghum genotypes under climate change-induced stresses such as CDHS could be attributed to rhizobacteria. Specific bacterial taxa are recruited in the rhizosphere environment of the plants for enhanced adaptation and crop productivity under CDHS conditions. Rhizobacteria can be utilized in plant breeding as targeted selection traits. Hence, more effort should be made to further exploit these beneficial plant–microbe interactions for enhanced sorghum productivity under abiotic stress conditions. In addition, evidence generated from this study may provide new plant breeding directions towards development of climate-resilient crop varieties through integration of rhizosphere microbiomes in plant selection processes.

## Figures and Tables

**Table 1:** List of *Sorghum bicolor* genotypes evaluated for rhizobacteria recruitment and GY performance at Chiredzi and Chisumbanje Research Station during the 2021–22 season under CDHS conditions.

Genotype Name	Origin	Status
SV4	Crop Breeding Institute, Harare, Zimbabwe	Released grain commercial variety [check]
ICSV111IN	ICRISAT—Hyderabad, India	Advanced pre-release line [Experimental]
CHITICHI	Chiredzi Community Seed Bank Masvingo, Zimbabwe	Local landrace variety [check]
MACIA	Seed Company of Zimbabwe Harare, Zimbabwe	Released grain commercial variety [check]
IESV91070DL	ICRISAT—Hyderabad, India	Advanced pre-release line [Experimental]
ASAREACA12-3-1	ICRISAT—Hyderabad, India	Advanced pre-release line [Experimental]

**Table 2:** MLR analysis of variance table based on a modelled relationship between rhizobacteria relative abundance data collected at post-flowering growth stage and *Sorghum bicolor* GY performance at the Chisumbanje Research Station, during the 2021–22 season under CDHS conditions.

Source	DF	Seq SS	Contribution (%)	Adj SS	Adj MS	F-Value	*p*-Value
Regression	2	0.54357	85.64	0.54357	0.27178	26.83	0.000
*Actinobacteriota Thermoleophilia*	1	0.40821	64.31	0.12504	0.12504	12.34	0.007
*Firmicutes Bacilli*	1	0.13536	21.33	0.13536	0.13536	13.36	0.005
Error	9	0.09116	14.36	0.09116	0.01013		
Total	11	0.63473	100.00				

Key: % contribution is the amount of variation in *Sorghum bicolor* GY performance (dependent variable) explained by a specific rhizobacterial taxa (independent variable) and the basis on which to rank their importance in enhancing plant adaptation to CDHS. Actinobacteriota Thermoleophilia is more important than *Firmicutes Bacilli*, explaining of 64.31% variation in sorghum GY performance compared to 21.33% for *Firmicutes Bacilli*.

**Table 3:** MLR analysis of variance table based on a modelled relationship between rhizobacteria relative abundance data collected at post-flowering growth stage and *Sorghum bicolor* GY performance at the Chiredzi Research Station, during the 2021–22 season under CDHS conditions.

Source	DF	Seq SS	Contribution (%)	Adj SS	Adj MS	F-Value	*p*-Value
Regression	2	6.395	52.23	6.395	3.1975	4.92	0.036
*Firmicutes Bacilli*	1	4.296	35.08	5.277	5.2769	8.12	0.019
*Actinobacteriota Actinobacteria*	1	2.099	17.14	2.099	2.0992	3.23	0.106
Error	9	5.850	47.77	5.850	0.6500		
Total	11	12.245	100.00				

Key: % contribution is the amount of variation in Sorghum bicolor GY performance (dependent variable), explained by a specific rhizobacterial taxa (independent variable), and the basis for ranking their importance in enhancing plant adaptation to CDHS. *Firmicutes Bacilli* is more important than Actinobacteriota Actinobacteria, which explained 35.08% variation in sorghum GY performance compared to the 17.14% for *Firmicutes Bacilli*.

**Table 4:** The across-site estimated genetic worth (VI) values of sorghum genotypes as determined through the Smith–Hazel analysis using relative abundance microbial data collected during the 2021–22 season under CDHS conditions.

Study Site	Genotype	Genetic Worth Index (V1)	GY (t/ha)
Chisumbanje Research Station	IESV91070DL	816.660	0.1910 ^c^
	ICSV111IN	601.739	0.9050 ^a^
	SV4	465.159	0.4150 ^bc^
	CHITICHI	463.827	0.6950 ^ab^
	ASARECA12-3-1	382.508	0.5000 ^bc^
	MACIA	22.790	0.6000 ^ab^
Chiredzi Research Station	ASARECA12-3-1	361.162	1.320 ^bc^
	IESV91070DL	271.417	1.335 ^bc^
	CHITICHI	251.939	0.480 ^c^
	SV4	205.087	3.495 ^a^
	MACIA	174.311	1.520 ^b^
	ICSV111IN	123.343	2.695 ^a^

Key: *Sorghum biolor* genotypes sharing the same letter(s) are not significantly different in their GY performance. Genetic worth (VI) of a *Sorghum bicolor* genotype is its estimated genetic makeup value which can be used by breeders to rank and select individuals.

**Table 5:** Ranking of *Sorghum bicolor* genotypes under solely conventional and conventional integrated with microbial diversity (i.e., genetic worth) selection criteria.

Conventional			Conventional Integrated with Microbial Diversity Data	
Genotype	Mean GY (t/ha)	Ranking	∑[Mean (GY + VI)]	Ranking
SV4	1.955	1	337.078	5
ICSV111IN	1.8	2	364.341	3
MACIA	1.06	3	99.6105	6
ASARECA12-3-1	0.91	4	372.745	2
IESV91070DL	0.763	5	544.8015	1
CHITICHI	0.5875	6	358.4705	4

Key: Conventional selection method refers to the ranking of sorghum bicolor genotypes based on their mean GY performance. Conventional integrated with microbial diversity data is a ranking based on the genotype’s summation of its average GY performance and mean genetic worth (VI) as determined through the Smith–Hazel Multi-Trait Stability Index (MTSI) analysis.

## Data Availability

The original contributions presented in this study are included in the article. Further inquiries can be directed to the corresponding author.

## Abbreviations

The following abbreviations are used in this manuscript:

MLR	Multiple Linear Regression
ANOVA	Analysis of Variance
MTSI	Multiple Trait Selection Index
ICRISAT	International Crops Research Institute for the Semi-Arid Tropics
CIMMYT	International Maize and Wheat Improvement Center
RCBD	Randomized Complete Block Design
CDHS	Combined Drought and Heat Stress
RCZ	Research Council of Zimbabwe
AIC	Akaike Information Criterion
BIC	Bayesian Information Criterion
DADA2	Divisive Amplicon Denoising Algorithm 2
PERMANOVA	Permutational Multivariate Analysis of Variance
out	Operational Taxonomic Unit
